# Carbon Domains on MoS_2_/TiO_2_ System via Catalytic Acetylene Oligomerization: Synthesis, Structure, and Surface Properties

**DOI:** 10.3389/fchem.2017.00091

**Published:** 2017-11-08

**Authors:** Sara Cravanzola, Federico Cesano, Fulvio Gaziano, Domenica Scarano

**Affiliations:** Department of Chemistry and Nanostructured Interfaces and Surfaces, Interdepartmental Centre and INSTM Centro di Riferimento, University of Turin, Turin, Italy

**Keywords:** hybrids, MoS_2_, TiO_2_, graphene, acetylene oligomerization, 2D materials, *in-situ* characterization, inorganic chemistry

## Abstract

Carbon domains have been obtained at the surface of a MoS_2_/TiO_2_ (Evonik, P25) system via oligomerization and cyclotrimerization reactions involved in the interaction of the photoactive material with acetylene. Firstly, MoS_2_ nanosheets have been synthesized at the surface of TiO_2_, via sulfidation of a molybdenum oxide precursor with H_2_S (bottom-up method). Secondly, the morphology and the structure, the optical and the vibrational properties of the obtained materials, for each step of the synthesis procedure, have been investigated by microscopy and spectroscopy methods. In particular, transmission electron microscopy images provide a simple tool to highlight the effectiveness of the sulfidation process, thus showing 1L, 2L, and stacked MoS_2_ nanosheets anchored to the surface of TiO_2_ nanoparticles. Lastly, *in-situ* FTIR spectroscopy investigation gives insights into the nature of the oligomerized species, showing that the formation of both polyenic and aromatic systems can be taken into account, being their formation promoted by both Ti and Mo catalytic sites. This finding gives an opportunity for the assembly of extended polyenic, polyaromatic, or mixed domains firmly attached at the surface of photoactive materials. The presented approach, somehow different from the carbon adding or doping processes of TiO_2_, is of potential interest for the advanced green chemistry and energy conversion/transport applications.

## Introduction

Carbon–based systems of different dimensionality, such as single/multiwalled carbon nanotubes, graphene-like, activated carbons, and carbon-based aerogels have attracted an increasing attention in the past and at the time of writing this paper. Recent works have shown that all these nanocarbons have distinctive properties, including electrical conductivity, chemical inertness, sorption, sensing, and biosensing (Cravotto et al., 2011; Cesano et al., 2012b, 2016; Holzinger et al., 2014; Badhulika et al., 2015; Li et al., 2015; Niu et al., 2015; Kotal et al., 2016). In the majority of cases, such properties can be better exhibited if nanocarbons are confined in another phase (i.e., polymers) (Wu et al., 2012; Cesano et al., 2013; Cravanzola et al., 2013; Niu et al., 2015), or are combined with other systems to form heterostructures (Wang et al., 2010; Ong, 2017; Rho et al., 2017), hybrids (Badhulika et al., 2015; Wang et al., 2016), composites (Bai and Shen, 2012), or simply when they are decorating the surface of inorganic solids (Wu et al., 2012; Cravanzola et al., 2016). Indeed, the different species of stacked 2D layers or heterostructures of different dimensionality have demonstrated to produce novel, unprecedented physical and chemical properties, or to increase the efficiency of the solar energy conversion, photocatalytic environmental remediation, sustainable fine chemistry thus creating new opportunities for device concepts and applications (Wu et al., 2012; Bubnova, 2016; Novoselov et al., 2016), which are for a fact all among the most challenging outlooks of the modern view of science.

Besides nanocarbons, prominent candidates for heterostructures and hybrid combinations belong to the family of semiconducting inorganic systems, such as nanostructured transition metal dichalcogenides (TMDs, i.e., MoS_2_, WS_2_) and oxides (i.e., TiO_2_, perovskites) (Rijnders, 2014; Duan et al., 2015). For instance, titanium dioxide polymorphs are by far the best candidates in some fields, offering an ideal platform for photocatalytic and catalytic applications (Cesano et al., 2012a; Topcu et al., 2016), but they have a relatively large bandgap (about 3–3.2 eV). Notwithstanding, feasible strategies may be adopted in harvesting the visible solar light. Among these, non-metal (e.g., N, C, S) (Cesano et al., 2008; Borges et al., 2016; Cravanzola et al., 2017) and metal doping (Uddin et al., 2008; Wang and Jing, 2014), sensitizer molecules (Yang et al., 2015), or quantum dots (Frame and Osterloh, 2010; Uddin et al., 2014) firmly anchored at the surface of the semiconductor or the formation of hybrid interfaces based on 2D materials (i.e., graphene, TMDs) (Bubnova, 2016), have been proposed as solutions. In this regard, several studies have demonstrated that single or few-stacked layers and/or small nanoparticles of 2D systems (i.e., graphene, TMDs) can be obtained from graphite and other bulk materials by adopting a top-down approach via the mechanical/chemical exfoliation (Coleman et al., 2011) or fragmentation (Muscuso et al., 2015) processes. Such layered systems can be thus transferred to surfaces to form van der Waals interfaces (Cravanzola et al., 2016; Novoselov et al., 2016).

In the present work, thin MoS_2_ slabs have been obtained from molybdenum oxide precursors in a sulfiding atmosphere (H_2_S) at the surface of TiO_2_, as already discussed for other oxides (SiO_2_, Al_2_O_3_, MgO) (Cesano et al., 2011) via a bottom-up approach, thus ensuring a good chemical interaction at the interface. In this domain, the *in-situ* structuring and self-assembling of carbon species at the MoS_2_/TiO_2_ surface are among the focus of this work, being stimulated by precedent studies reporting the facile acetylene interaction occurring at room temperature at the surface of pure TiO_2_. Such interaction, entailing a complex set of reactions, is responsible for the formation of polyaromatic domains or polyacetylenic moieties firmly grafted at the surface of the solid (Sakata et al., 1991; Mino et al., 2012; Biedrzycki et al., 2014; Jain et al., 2014). In the present paper, the role of MoS_2_ in affecting the C_2_H_2_ interaction at the surface of MoS_2_-decorated TiO_2_, is investigated by microstructural (X-ray diffraction), microscopic methods (scanning and transmission electron microscopies) and spectroscopies (i.e., infrared, Raman, and UV-vis). Notably, the irreversible products entailed in the set of oligomerization reactions can be governed by the temperature and time, which help in the control of the aromatic or double-bonded polyconjugated domains formation. Our method, aiming to perform a molecular self-assembly approach to nanomaterials, outlines as far as possible the direct production of strongly anchored species to the photoactive material, such as graphene-like or polyacetylene chains, both interesting for applications in the field of catalysis for advanced green chemistry, moving from H_2_ production activity, to hydrodesulfurization or to organic pollutants photodegradation reactions.

## Materials and methods

### Preparation of MoO_x_/TiO_2_ samples

A water solution of ammonium heptamolybdate powder (AHM, Merck) was added drop by drop to 2 g TiO_2_ (P25, Evonik), by following a wet impregnation method. In order to remove the solvent, the impregnated powder was then dried in air, overnight. The final concentration of Mo was about 3 wt%. A more concentrated AHM/TiO_2_ sample (Mo 50 wt%) was also obtained to be used as a reference material for XRD measurements. A preliminary air thermal treatment step in a muffle furnace (673 K, 12 h) was adopted to better decompose the AHM salt and to remove the developing ammonia and water.

Samples activation and sulfidation: MoO_x_/TiO_2_ samples, in the form of self-supported pellets, were activated under dynamic vacuum at 673 K for 30 min, and then twice oxidized in an oxygen atmosphere (40 Torr) at 673 K, for 30 min. By keeping the same temperature, the oxidized samples were sulfided in the H_2_S atmosphere (30 Torr) for 1 h, then outgassed. Pellets were successively further sulfided, following the same procedure. All the steps were followed by *in situ* FTIR, to avoid the re-exposure of the samples to air.

### *In situ* C_2_H_2_ oligomerization

The C_2_H_2_ interaction at the surface of both MoO_x_/TiO_2_ and MoS_2_/TiO_2_ samples was investigated in such a way to highlight the role of molybdenum and sulfur during the oligomerization reaction. The adopted procedure can be described as follows: (i) preliminary activation of the samples at 673 K inside an IR cell; (ii) oxidation at the same temperature, as described above. After that, the samples were cooled down and outgassed. Then acetylene (120 Torr) was dosed on the samples at 293 K. The reaction was followed by *in situ* IR spectroscopy for 30 min, in such a way to investigate the evolution of the products in time. Thereafter, to understand the effect of the temperature, the samples were heated at 373 K for 30 min, cooled down to 293 K, and investigated by IR spectroscopy. The same procedure was also repeated at 423 K.

### Samples characterization

Materials were investigated at each step of preparation, before (MoO_x_/TiO_2_, MoS_2_/TiO_2_) and after C_2_H_2_ oligomerization (hereafter *p*-C_2_H_2_/TiO_2_ and *p*-C_2_H_2_/MoS_2_/TiO_2_, respectively). X-ray diffraction patterns were collected by means of a PANalytical PW3050/60 X'Pert PRO MPD diffractometer working with a Ni-filtered Cu anode, working in a Bragg-Brentano geometry and using the spinner mode. The morphology of samples was investigated by means of a Zeiss Evo 50 SEM instrument operating at 30 kV, equipped with an energy dispersive X-ray (EDAX) detector. Transmission electron microscopy images were acquired with a JEOL 3010-UHR instrument operating at 300 kV, equipped with a 2 × 2 k pixels Gatan US1000 CCD camera. Samples were deposited on copper grids covered by a lacey carbon-based film. Reciprocal lattices were simulated by means of CaRIne Crystallography 3.1 software. Raman spectra were obtained by using a Renishaw Raman InVia Reflex spectrophotometer equipped with an Ar^+^ laser emitting at 442 and 514 nm, using a static or a rotating configuration. UV-Vis spectra were collected by using a Varian Cary 5000 UV-vis-NIR spectrophotometer, equipped with a reflectance sphere. Owing to their strong optical absorption, the samples were diluted in BaSO_4_ powder and investigated in air. N_2_ adsorption/desorption experiments have been performed at 77 K (Micromeritics ASAP 2020 instrument) to determine the Brunauer–Emmett–Teller (BET) surface area and micropore volume (t-plot method). Before the surface area determination, samples were outgassed at RT overnight. Pore size distributions (PSDs) were carried out by means of a non-negative least squares fitting on the absorption isotherm data by applying the Density Functional Theory (DFT) method (N_2_-DFT model, slit geometry) by means of the MicroActive Datamaster 5 software (Micromeritics). Microporous (S_micro_) and mesoporous (S_meso_) surfaces were obtained from t-plot and from S_meso_ = S_tot_-S_micro_, respectively. Finally, FTIR spectra of CO adsorbed at the surface of the samples were obtained at 77 K in an IR cell designed for the liquid N_2_ flowing, and recorded by means of a Bruker IFS-28 spectrometer, equipped with a MCT detector, having a resolution of 4 cm^−1^ (64 interferograms were recorded and averaged for each spectrum). The spectra were acquired in the 4,000–400 cm^−1^ interval where the fundamental vibration modes are observed. The same instrument was used to follow the *in-situ* investigation of oligomerization reactions, by using a room temperature IR cell. Samples for FTIR measurements were in the form of thin self-supported pellets.

## Results and discussion

### Structure and morphology of the samples: XRD and HRTEM analyses

**Figure 1:**
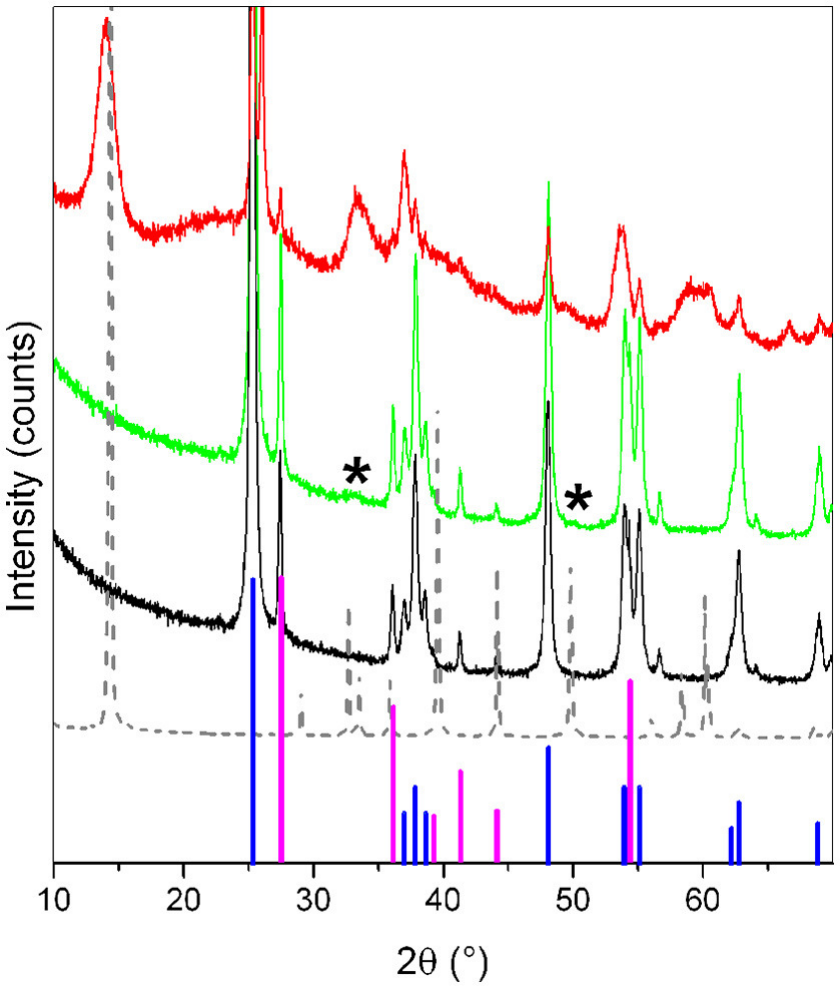
XRD patterns of TiO_2_ (black line), of MoS_2_/TiO_2_ (Mo 3 wt.%) (green line), of the more concentrated MoS_2_/TiO_2_ sample (Mo 50 wt.%) (red line). and of pure bulk MoS_2_ used as a reference material (gray dotted line) Asteriks (^*^) highlight two weak diffraction features typical of MoS_2_. Anatase (PDF card # 21-1272) and rutile (PDF card # 21-1276) phases (blue and magenta lines, respectively) are shown for comparison.

XRD patterns of the samples are shown in Figure 1. From these, information on the crystalline structure was obtained. MoS_2_/TiO_2_ (green line) is compared to pure TiO_2_ (black line), which shows the usual crystalline features of anatase and rutile phases (PDF card # 21-1272 and # 21-1276, respectively), as highlighted by blue and magenta lines (anatase and rutile, respectively).

No significant differences are emerging from the comparison between patterns of MoS_2_/TiO_2_ and TiO_2_ as such. In more details, the typical XRD fingerprints of MoS_2_ (gray dotted line) are absent or very weak, owing to the low concentration of Mo in the sample, as highlighted by the two asterisks (^*^) at 2θ = 33° and 49° in Figure 1. According to the XRD pattern of the more concentrated MoS_2_/TiO_2_ sample (red line), these two features are associated with the diffraction of (101) and (105) planes of MoS_2_ (PDF card # 37-1492), respectively, being the only evidence of MoS_2_ presence in the sample (*vide infra*). In point of fact, notice that the (002) plane diffraction feature of MoS_2_ along c-axis (related to the slab stacking) is absent, as well as no evidence of XRD peaks associated with the MoO_x_ phases (here not shown for the sake of simplicity), is highlighted. This means that the sulfidation process after contacting the TiO_2_ powder with H_2_S at 673 K for 2h is completed (Cesano et al., 2011).

The obtained MoS_2_/TiO_2_ samples before and after the interaction with C_2_H_2_ have been SEM imaged (Supplementary Figure 2). No significant differences with respect to the TiO_2_ P25 nanopowder are observed for both samples at the adopted resolution. To give more insights, TEM investigations have been performed for both the samples.

**Figure 2:**
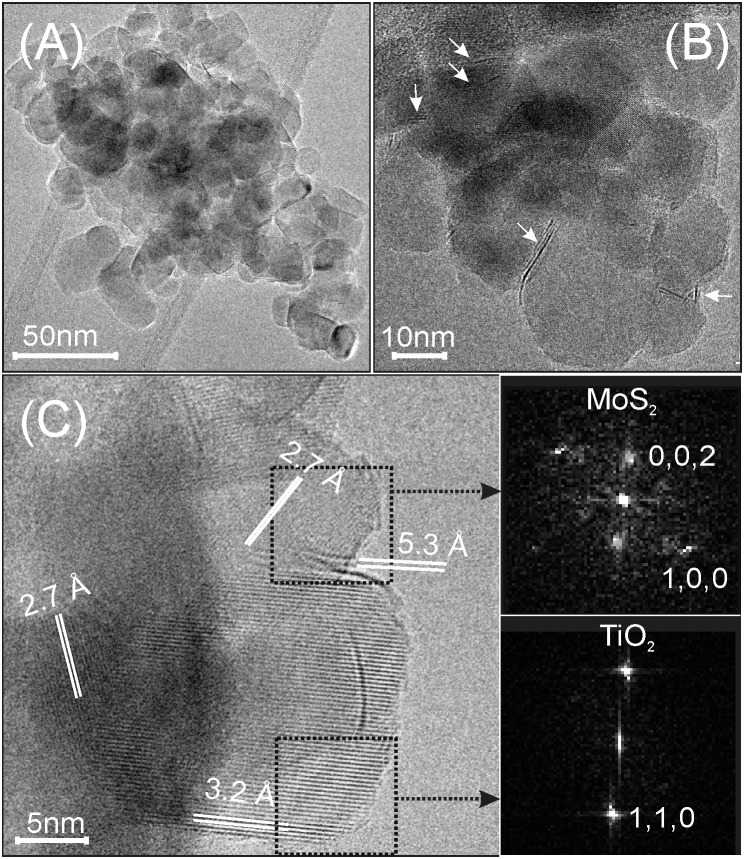
**(A,B)** TEM images, acquired with different resolutions, of MoS_2_ decorated TiO_2_ nanoparticles. The white arrows indicate mono- and few-layer MoS_2_ slabs; **(C)** HRTEM image of thin particles, showing the MoS_2_ (100) and (002) interference fringes. Fast-Fourier-transform (FFT) images of the two selected regions in **(C)** (insets on the right side).

In Figures 2A,B MoS_2_/TiO_2_ nanoparticles before the interaction with acetylene are TEM imaged and can be compared with TiO_2_ P25 (Cravanzola et al., 2017). It can be observed that 1L, 2L, and few-layer MoS_2_ slabs (see arrows) exposing (002) planes (c-axis) are decorating the surface of TiO_2_ nanoparticles.

Most of the thin MoS_2_ sheets are 3–10 nm in size, but larger slabs are also present. From the HRTEM image (Figure 2C), a TiO_2_ nanoparticle 20 nm in size exposes 0.32 nm spaced lattice fringes, which correspond to (110) planes of rutile (from tabulated values, PDF card # 21-1276, see the fast-Fourier transform, FFT image in the bottom inset). Moreover, a curved 7–8 nm long feature, which is superimposed to the TiO_2_ nanoparticle lattice fringes along a nearly perpendicular direction, is also observed (Figure 2C). This is most likely associated with a 1L-MoS_2_ sheet. Another MoS_2_ slab (2–3 layers thick) decorates the surface of the rutile nanocrystal, as well. In this region, 0.27 nm spaced lattice fringes, attributed to the (100) planes of MoS_2_ are in agreement with the arrangement and spacing of the bright spots shown in the FFT image (Figure 2C top inset) (Muscuso et al., 2015). These images are also clearly indicative of the effectiveness of the sulfidation process, that causes increase of defects at the surface (Cravanzola et al., 2017).

**Figure 3:**
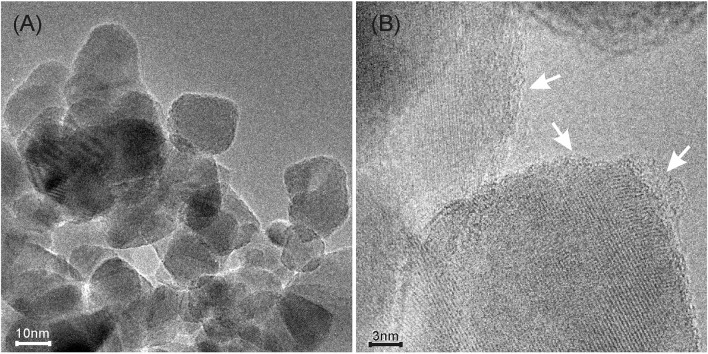
**(A)** TEM image of *p*-C_2_H_2_/MoS_2_/TiO_2_ sample; **(B)** HRTEM image of the same sample showing the carbon based envelope originated after exposure to C_2_H_2_ (highlighted by white arrows).

The MoS_2_/TiO_2_ sample has been also TEM and HRTEM imaged after the C_2_H_2_ oligomerization at RT for 30′ (Figure 3).

From the images, it is clear that the TiO_2_ nanoparticles are covered by a thin layer after the interaction with C_2_H_2_. The nature of this layer will be discussed in the following.

### Surface properties and porosity of the samples: Raman, UV-vis and volumetric analyses

**Figure 4:**
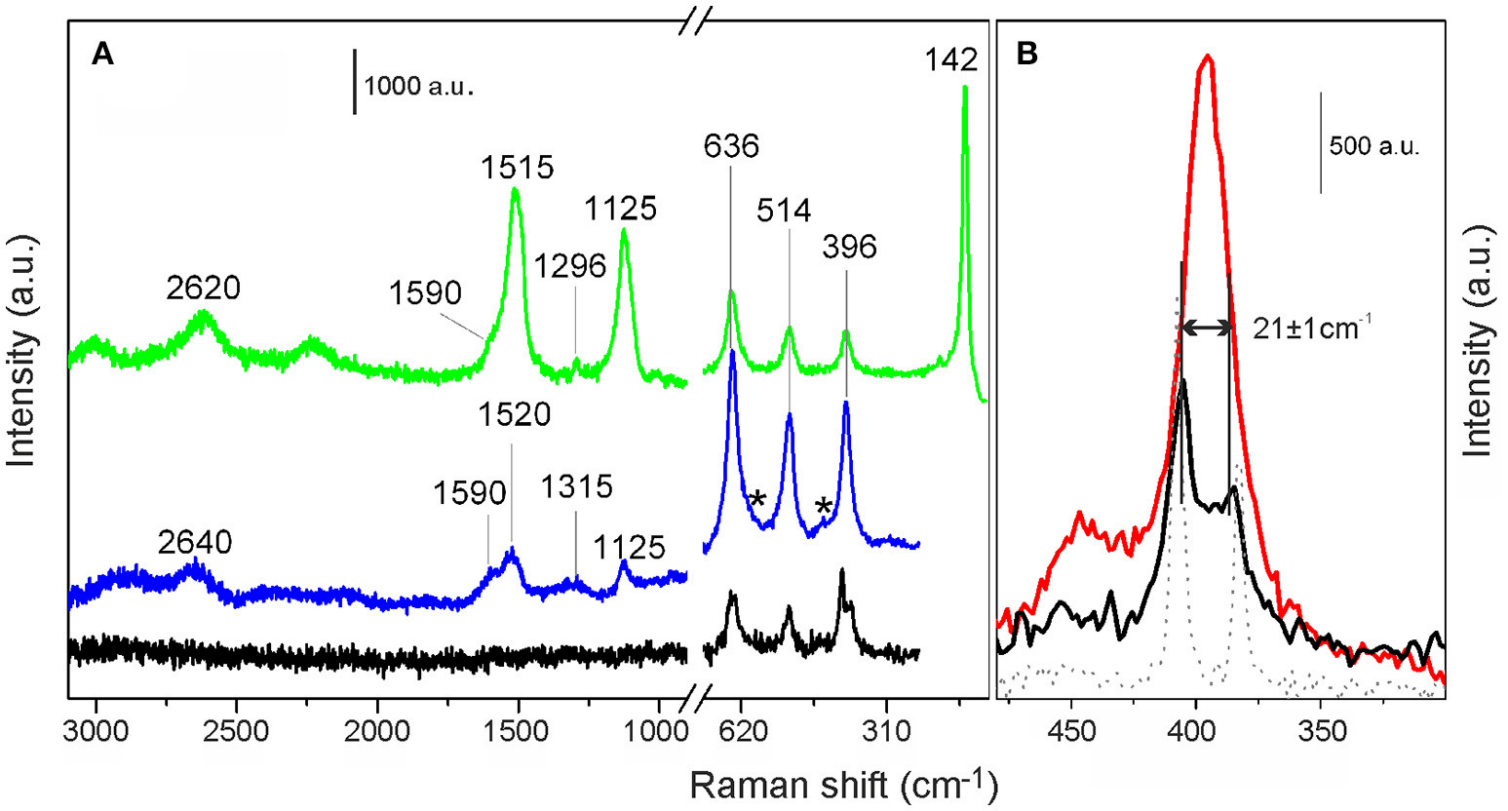
**(A)** Raman spectra of *p*-C_2_H_2_/MoS_2_/TiO_2_ recorded with the 442 nm and 514 nm laser lines, (blue and green curves, respectively). Raman spectrum of MoS_2_/TiO_2_, recorded with the 442 nm laser line (black curve) is shown for comparison. **(B)** Enlarged view in the 400–500 cm^−1^ spectral range, of MoS_2_/TiO_2_ (black curve), pure TiO_2_ (red curve) and pure MoS_2_ (gray dotted line) spectra, acquired with the 442 nm laser line.

Raman spectra, acquired with the 442 nm laser line, of MoS_2_/TiO_2_ (black curve) and of *p*-C_2_H_2_/MoS_2_/TiO_2_ (blue curve) are compared in Figure 4A and Supplementary Figure 3.

In the 750–250 cm^−1^ range, the typical modes of TiO_2_ are observed for both the investigated samples. More in detail, as well described in literature (Cesano et al., 2008; Cravanzola et al., 2017), the bands at 396, 514, and 636 cm^−1^ are attributed to the B_1g_, A_1g_, and E_g_ Raman active modes, respectively, of the TiO_2_ anatase phase (Ohsaka et al., 1978; Ma et al., 2007). Furthermore, the small peak at 444 cm^−1^ and the shoulder at 608 cm^−1^ (labeled by stars) are due to the E_g_ and A_1g_ modes of the rutile phase (Ma et al., 2007). It is noteworthy that the expected peak at 142 cm^−1^ of anatase is here absent, because a filter has been used in the experimental setup.

Moving to the 3,000–1,000 cm^−1^ region, no spectral modes are observed for MoS_2_/TiO_2_ sample (black curve) as expected, then the following discussion will be focused on the effects of acetylene oligomerization on MoS_2_/TiO_2_ system (blue curve).

It is well-known that the carbon-carbon skeletal vibrations of polyenes usually are observed in the 1,600–1,500 cm^−1^ range and at about 1,100 cm^−1^, being their precise position dependent on the number of double bonds in the chain molecules (Harada et al., 1980; Rives-Arnau and Sheppard, 1980; Pena-Alvarez et al., 2016). Precisely, the bands observed after dosing acetylene on MoS_2_/TiO_2_ at 1,520 and 1,125 cm^−1^ (blue curve), can be associated in this specific case with the formation of polyenes chains.

Going into more details, the peaks at 1,520 and at 1,125 cm^−1^ are assigned to the polyconjugated C = C double bond stretching mode ν_1_ and to the mixed mode ν_3_ of the –CH bending and C–C single bond stretching vibrations of trans-polyacetylene, respectively (Harada et al., 1980; Pena-Alvarez et al., 2016). It is known that the frequency and the intensity values change with the laser excitation, as confirmed by Harada et al. (1980). Furthermore, the laser excitation dependence and sideband shifts should be taken into account considering resonant Raman scattering effects (Heller et al., 2015). In our experiment (λ _ex_ = 422 nm), the obtained results are in agreement with the series in the 350.7 < λ _ex_ < 647.0 nm range, observed by some authors (Harada et al., 1980; Rives-Arnau and Sheppard, 1980; Castiglioni et al., 2004). Even so, when Raman spectra of *p*-C_2_H_2_/MoS_2_/TiO_2_ acquired with 442 nm (blue curve in Figure 4A) and 514 nm (green curve) laser lines are compared, remarkable changes are detectable. Some more, the number of conjugated C = C bonds can be roughly determined, as the frequency shifts is a function of the conjugation length. Notice that the resonant Raman experiment preferentially excites chains with energy gap close to the laser line energy. As far as it goes, a conjugation number of about 7–8 can be hypothesized, in agreement with data reported in literature (Schaffer et al., 1991; Castiglioni et al., 2004). Furthermore, it is noteworthy that from Raman spectra other two features can be highlighted. Firstly, the shoulder at 1,590 cm^−1^ and the small peak at 1,320 cm^−1^ can be assigned to the typical G mode of the sp^2^ carbon networks and to the disorder induced D bands of graphitic materials, respectively (Pimenta et al., 2007; Cesano et al., 2008). Lastly, the band at 2,640–2,620 cm^−1^ deserves also attention. According to the literature, the band could be ascribed to the 2D second order overtone of the D band of few-layer graphene (Ferrari and Basko, 2013). Notice that, for graphene based systems, the 2D fingerprint is usually much more intense, even four times, than the G band, being its intensity and width very sensitive to the stacking order of the graphene sheets along the c axis (Ferrari et al., 2006; Pimenta et al., 2007). Without going into details, from the position and shape of the G (1,590 cm^−1^) and 2D (2,640–2,620 cm^−1^) modes (blue and green lines in Figure 4A) the formation of graphene and/or graphitic domains can be inferred.

In conclusion, the formation of polyenes and chains with conjugated double bonds, together with very thin graphenic domains, containing a considerable number of condensed rings, can be assumed. This is in agreement with the FTIR results (*vide infra*) and with the computational data (Castiglioni et al., 2004).

Moving to the 500–400 cm^−1^ spectral range (Figure 4B), the Raman spectra of MoS_2_/TiO_2_ (black curve) and of TiO_2_ as such (red curve), both acquired with the 442 nm laser line, are compared. It can be noticed that for MoS_2_/TiO_2_ system, the feature centered at 396 cm^−1^, is split into two main components at 405 cm^−1^ and at 384 cm^−1^, which can be assigned to the A_1g_ and ${\text{E}}_{2\text{g}}^{1}$ first-order Raman active modes of MoS_2_ (Cravanzola et al., 2015). As the difference in the frequency of A_1g_ and ${\text{E}}_{2\text{g}}^{1}$ modes is indicative of the MoS_2_ slabs thickness, the observed separation of 21 ± 1 cm^−1^ is, in our case, representative of a stacking number of 2 ± 1 layers (Lee et al., 2010; Cesano et al., 2011), thus confirming the results obtained by TEM images.

**Figure 5:**
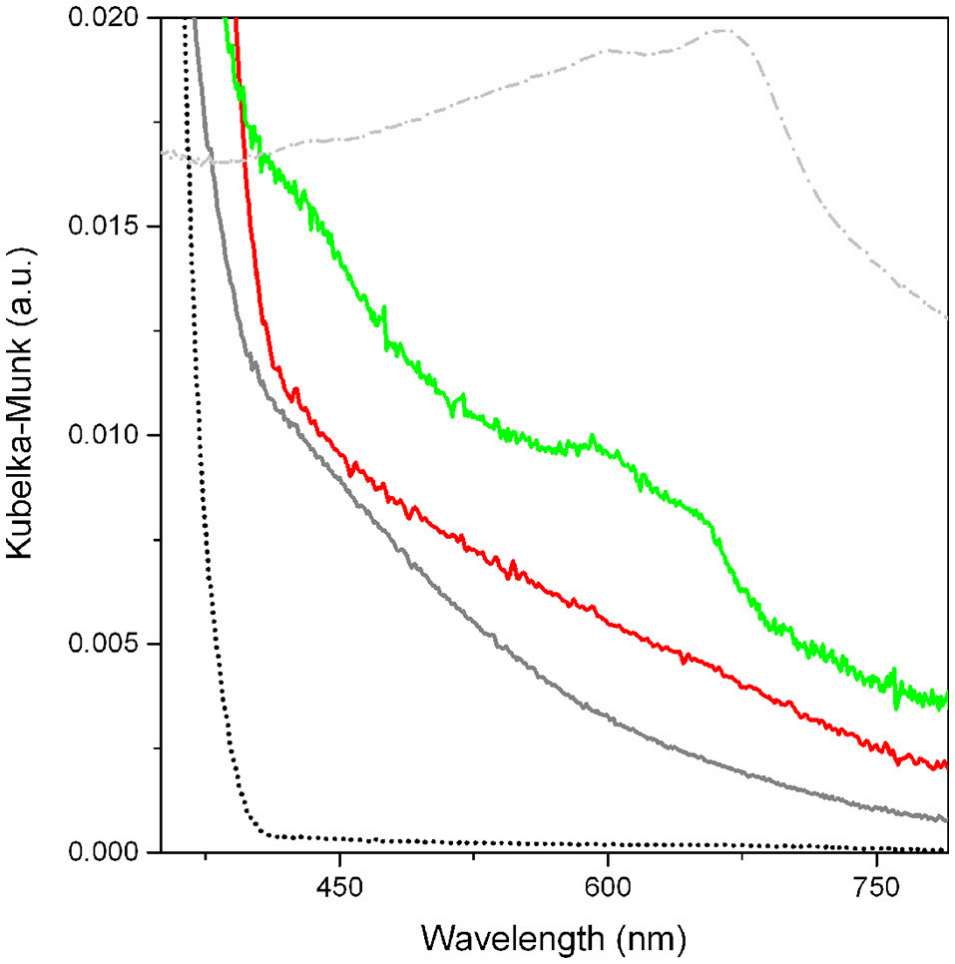
UV-vis spectra of TiO_2_ used as a reference (black dotted curve), *p*-C_2_H_2_/TiO_2_ (gray curve), *p*-C_2_H_2_/MoS_2_/TiO_2_ (red curve), MoS_2_/TiO_2_ (green curve), and MoS_2_ (gray dotted line).

UV-vis spectra of *p*-C_2_H_2_/TiO_2_, *p*-C_2_H_2_/MoS_2_/TiO_2_, MoS_2_/TiO_2_, TiO_2_, and MoS_2_ used as a reference, are shown in Figure 5.

From the comparison between spectra of *p*-C_2_H_2_ MoS_2_/TiO_2_ (red curve) and TiO_2_ as such (black dotted curve), a wide absorption in the 400–800 nm range is observed for the C_2_H_2_ oligomerized on pure TiO_2_. Even so, this phenomenon is likely due to the formation of conjugated double bond species absorbing light in the visible range. It is hypothesized that, owing to its high intensity and width, the observed absorption could be described with the formation of conjugate systems with a different number of double bonds.

The results are confirmed by the intense blue color, stable for days in the open air, of the C_2_H_2_-contacted self-supporting pellet. Hence, the presence of irreversible species, tightly anchored to the surfaces of TiO_2_, is demonstrated (Jain et al., 2014). Some more, the typical manifestations of the MoS_2_, are well illustrated in the UV-Vis spectrum of MoS_2_/TiO_2_ (green curve of Figure 5), if compared to bulk MoS_2_ (gray dotted curve) The modes at about 680 nm and at 600 nm have been explained with A and B excitonic transitions, respectively, whose separation in energy can be related to the spin-orbit splitting at the top of the valence band at the K point of the Brillouin zone. The broad envelope in the 350–450 nm interval can be explained with other typical MoS_2_ C and D excitonic transitions, associated with a threshold at about 500 nm. The presence of these typical features, together with the continuous absorption extended over the entire Vis-NIR range, is consistent with the reduction of Mo^6+^ to Mo ^(6−x)+^ (*x* = 1,2) (Signorile et al., 2015), and is a further proof of the formation of thin MoS_2_ after H_2_S dosage on the MoO_x_/TiO_2_ support (Muscuso et al., 2015). Moreover, moving to the *p*-C_2_H_2_/MoS_2_/TiO_2_ system (red curve), the extended features previously assigned to the C_2_H_2_ oligomerization products, obscure the typical MoS_2_ modes.

**Figure 6:**
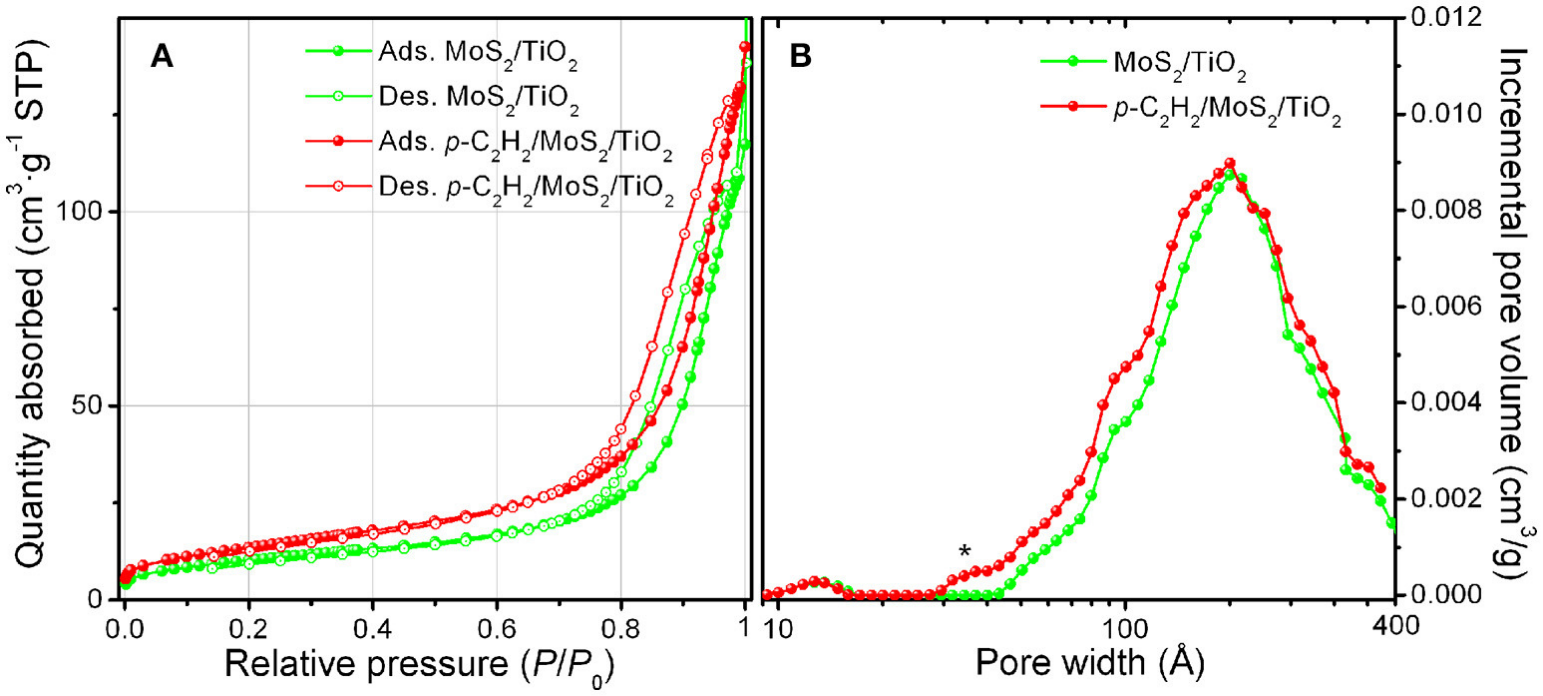
N_2_-absorption/desorption isotherms **(A)** and pore size distribution **(B)** of MoS_2_/TiO_2_ sample (green curves) and *p*-C_2_H_2_/MoS_2_/TiO_2_ as obtained after C_2_H_2_ treatment. The asterisk in **(B)** highlights a weak additional contribution of small pores, 30–40 Å in size, for *p*-C_2_H_2_/MoS_2_/TiO_2_ (red curve).

Volumetric N_2_ adsorption/desorption isotherms and pore size distributions of MoS_2_/TiO_2_ and of *p*-C_2_H_2_/MoS_2_/TiO_2_ are shown in Figure 6A. BET surface area (S_BET_), microporous (S_micro_) and mesoporous (S_meso_) surface area properties of samples are reported in Table 1. Both isotherms are of type IV with hysteresis loops, thus indicating the mesoporous character of samples and the negligible contribution of small micropores. This remark is confirmed by the PSDs reported in Figure 6B. More in details, a very broad family of pores in the 40–400 Å range is shown for both the samples together with the appearance of a weak additional contribution of small pores 30–40 Å in size, which does not alter the porosity character of the sample after the polymerization. A small increment of the SSA after the oligomerization of C_2_H_2_ is also observed, thus indicating the formation of a sort of scaffold, mesoporous in character, around the TiO_2_ nanoparticles.

**Table 1:** Surface area and porosity properties.

**Sample**	**S_BET_ (m^2^/g)**	**S_micro_ (m^2^/g)**	**S_meso_ (m^2^/g)**
MoS_2_/TiO_2_	37	-	37
*p*-C_2_H_2_MoS_2_/TiO_2_	48	-	48

### *In-situ* C_2_H_2_ oligomerization and surface properties: FTIR spectroscopy

A more complete understanding of the nature of the catalytic sites and of oligomerized species occurring in the oligomerization processes can be achieved by means of *in-situ* FTIR spectroscopy.

**Figure 7:**
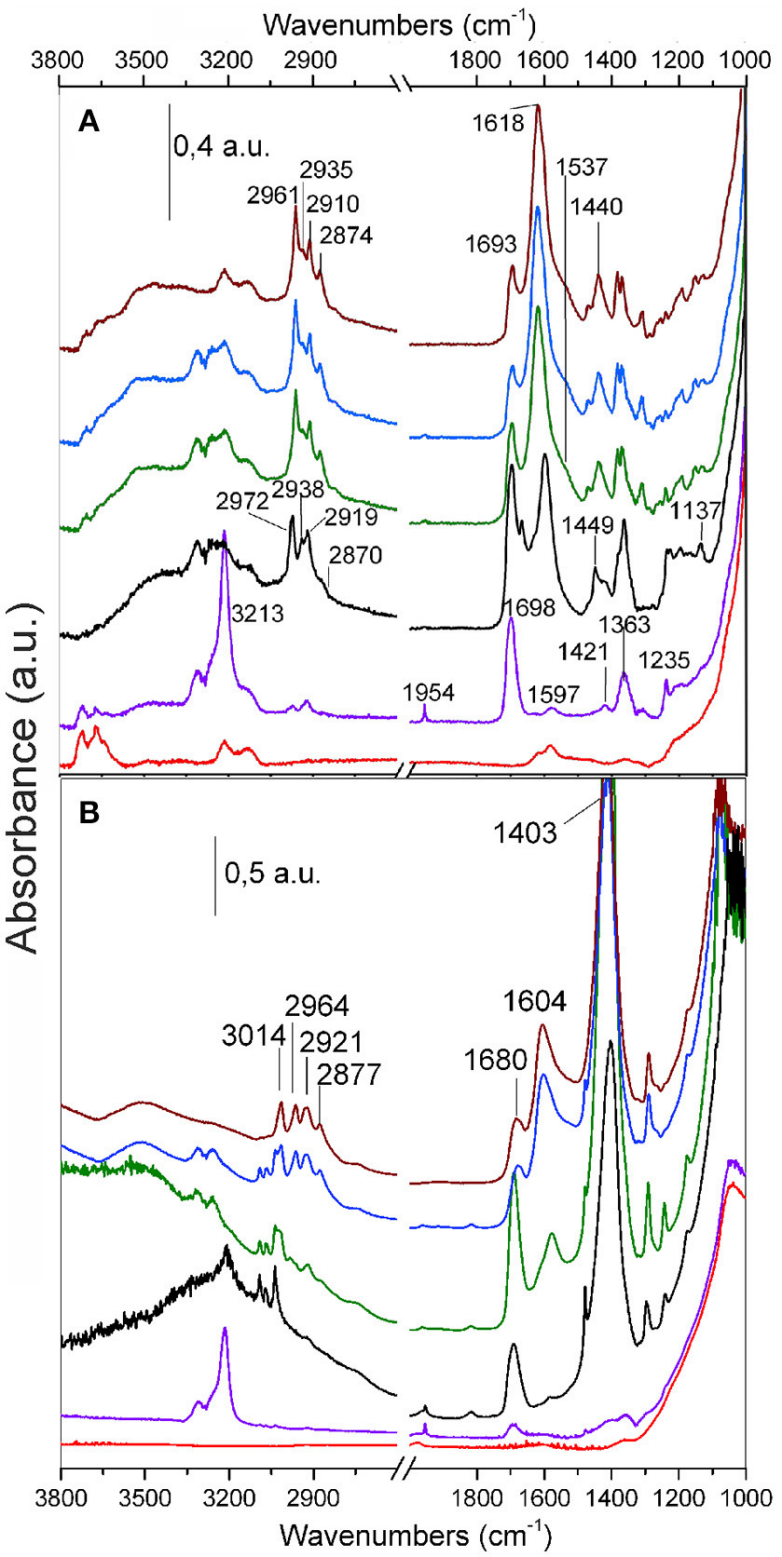
FTIR spectra of C_2_H_2_ (120 Torr) adsorbed on TiO_2_ **(A)** and MoS_2_/TiO_2_ **(B)**. The effect of the increasing contact time and temperature is shown: background (red curves); 1 and 30 min contact time at RT (purple and black curves, respectively); 30 min contact time at 373 and 423 K (green and blue curves, respectively); outgassing at RT (brown curves).

FTIR spectra of C_2_H_2_ dosed on TiO_2_ and on the MoS_2_/TiO_2_ sample are compared in Figures 7A,B.

A remark about the early interaction of acetylene with TiO_2_ (Figure 7A), as obtained after 1 min of contact time at RT (purple curve), can be made. The main developing bands at 1,954 and 3,213 cm^−1^ are due to the –C=C– and =CH modes of adsorbed acetylene, whose formation is accompanied by the complete erosion of the OH groups features with maxima at 3,716 and 3,667 cm^−1^, together with the developing of a broad absorption covering the entire 3,700–3,000 cm^−1^ range. This fact can be described with the occurrence of hydrogen bonding interactions, which involve not only the acetylene molecules, but also the reaction products (Mino et al., 2012).

By increasing the acetylene contact time (black curve) and reaction temperature (green and blue curves), an even more complex evolution of the spectra is observed, that is: (i) the decreasing in intensity of bands at 3,213 and 1,954 cm^−1^ (adsorbed acetylene), ii) the increasing of absorption bands at 1,698, 1,597, 1,449, 1,421, 1,363, 1,235, 1,196, and 1,137 cm^−1^ (i.e., initial products), iii) a quartet of bands (2,972 cm^−1^, 2,938 cm^−1^, 2,919 cm^−1^, and shoulder at 2,870 cm^−1^) is for a fact associated with the stretching vibrations of –CH_x_ groups (aliphatic molecules owing to saturated C-H, such as CH_2_ and CH_3_). More in details the peak at 1,698 cm^−1^ and the features gradually developing at 1,666 cm^−1^, 1,630 cm^−1^ (shoulder), 1,625 cm^−1^ (shoulder), and 1,597 cm^−1^ are in the region where conjugated double bonds are found, although they do not find exact correlation with those of the polyacetylenic species, reported by other authors (Rives-Arnau and Sheppard, 1980). Moreover, the quartet of bands at 2,972, 2,938, 2,919, and 2,870 cm^−1^ (shoulder) increases and shifts to 2,961, 2,935, 2,910, and 2,874 cm^−1^. The doublet at 2,961–2,910 cm^−1^ is due to aliphatic –CH_3_ groups (Groppo et al., 2006), while the assignment of the pair at 2,935 and 2,874 cm^−1^, is more uncertain. In particular the former could be associated with the stretching of –CH_2_ groups, whereas the 2,874 cm^−1^ band may belong to –CH of alkanes (Mistry, 2009).

It is noteworthy that the absorption intensities change with the contact time, becoming predominant with longer reaction time. This is a suggestion that more and more complex oligomerization products, containing an increasing number of conjugated double bonds, are progressively formed.

Along with the interaction of acetylene with MoS_2_/TiO_2_ surfaces (Figure 7B), a large and very intense peak in the 1,470–1,350 cm^−1^ range, centered at 1,403 cm^−1^, could be assigned to the overlapping of in- plane bending modes of –CH_x_ grown on defective sites of MoS_2_ layers covering TiO_2_ surfaces (Mistry, 2009). By increasing the contact time (black curve) at RT, a triplet of bands at 3,092, 3,068, and 3,035 cm^−1^ is developing, associated to the stretching vibrations of –CH groups belonging to aliphatic and/or aromatic cyclic molecules (Mistry, 2009).

On the other hand, by increasing the temperature, the intensity of this triplet of bands is decreasing, thus favoring the developing of a new quartet at 3,014, 2,964, 2,921, and 2,877 cm^−1^ (green and blue curves). The peak at 3,014 cm^−1^ is due to CH stretching of alkenes (Mistry, 2009), the doublet at 2,964–2,921 cm^−1^ is due to aliphatic –CH_3_ groups, while the 2,877 cm^−1^ band may be due to –CH of alkanes, respectively (Colthup et al., 1991; Mistry, 2009). It is noteworthy that a further outgassing of the sample at RT under high vacuum (brown curve) causes the disappearance of the bands of adsorbed acetylene but does not affect those related to the reaction products.

As the positions of CH_x_ stretching vibrational modes in the infrared spectra are strictly associated with the nature of the carbon bonding (C-sp^2^: 3,100–3,000 cm^−1^; C-sp^3^: 3,000–2,800 cm^−1^) at this point, it is important to recall that from FTIR spectra the contribution of species containing C-sp^2^ and C-sp^3^ bondings can be evaluated (Molpeceres et al., 2017). In this regard the role played by MoS_2_/TiO_2_ surfaces upon the thermal treatment in the conversion from insaturated (C-sp^2^) to saturated species (C-sp^3^) can be evidenced.

The nature of the chemical status of the other elements is discussed in the following.

### Interaction of CO at the surface of TiO_2_, MoS_2_/TiO_2_, and *p*-C_2_H_2_/MoS_2_/TiO_2_

**Figure 8:**
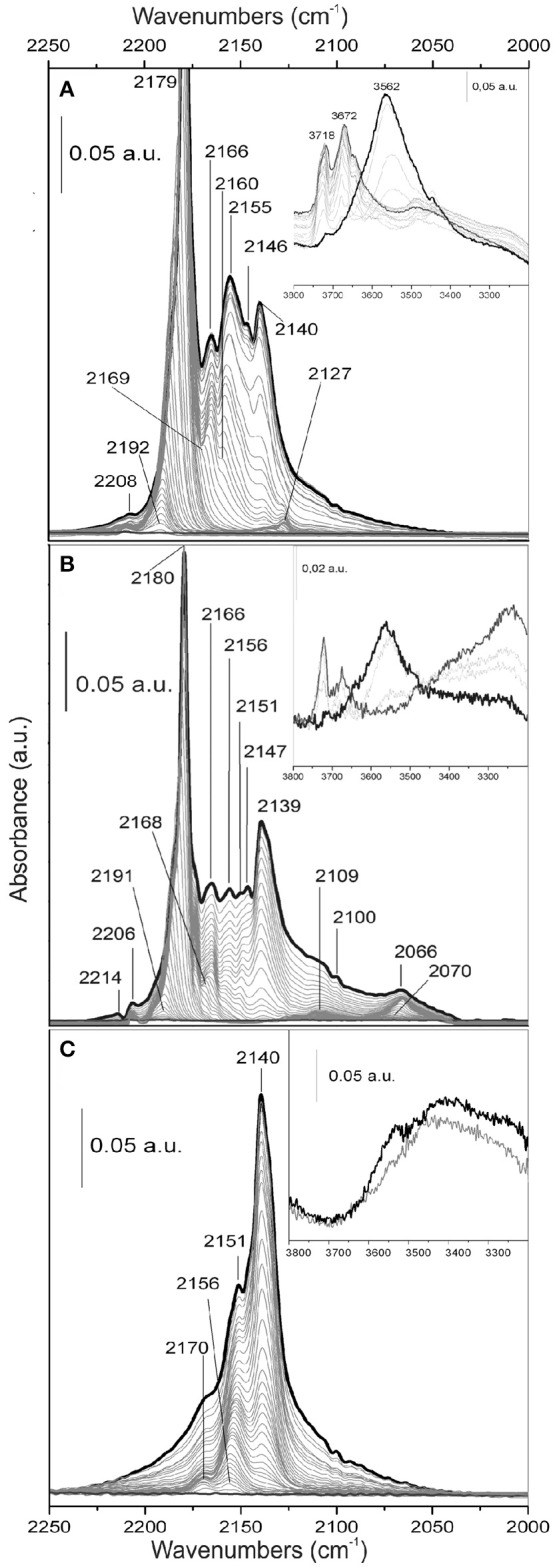
FTIR spectra of CO adsorbed at 77 K on the surface of: **(A)** TiO_2_; **(B)** MoS_2_/TiO_2_; **(C)** *p*-C_2_H_2_/MoS_2_/ TiO_2_. In the insets, the hydroxyl groups region is shown.

FTIR spectra of CO adsorbed at 77 K on TiO_2_, MoS_2_/TiO_2_ and on *p*-C_2_H_2_/MoS_2_/TiO_2_ are compared (Figure 8).

From a first analysis, the main difference between the FTIR spectra of CO adsorbed on TiO_2_ and on MoS_2_/TiO_2_ surfaces is a general decreasing of the main features intensities (Figures 8A,B). In point of fact, if Figure 8A is taken into consideration, the intense main peak centered at 2,179 cm^−1^ can be assigned to parallel CO oscillators, interacting with 5-fold coordinated Ti^4+^ sites located on flat (101) surfaces (Mino et al., 2012), while the one at 2,155 cm^−1^ is associated with CO molecules interacting with hydrogen bond to residual OH groups, as well discussed by some authors (Spoto et al., 1990; Bordiga et al., 1993; Martra, 2000; Mino et al., 2012). Notice that the bands at 3,719 and 3,672 cm^−1^, due to the stretching mode of hydroxyl groups present on the surface, (inset of Figure 8A) are shifted to lower frequency after CO adsorption, thus giving rise to a broader and more intense feature centered at 3,562 cm^−1^. The original spectral profile of the hydroxyl groups is fully restored by decreasing the CO gas pressure together with the progressively disappearance and shifting to 2,160 cm^−1^ of the 2,155 cm^−1^ band. These two events remark that the complete desorption of CO from OH groups occurs (Martra, 2000).

The sharp band at 2,140 cm^−1^ is also associated to physically adsorbed CO forming a multilayered surface when liquid N_2_ temperature brings CO to “liquid-like” state (Bordiga et al., 1993). Furthermore, the adsorption band at 2,146 cm^−1^ gradually shifts upward and merges into the band at 2,155 cm^−1^ by decreasing CO coverage, which is associated with the interaction of CO with facelets of the rutile phase (Mino et al., 2012). The weak band at 2,166 cm^−1^ can be assigned to CO adsorbed on Ti acidic Lewis centers located on flat (001) surfaces, where Ti centers along Ti-O rows are strongly bound to two O anions, causing at these sites a more screened electrostatic potential and, therefore, a reduced acidity. The weak band at 2,208 cm^−1^ is related to CO adsorbed on highly acidic Ti Lewis sites exhibiting very low coordination (3- and 4-folds coordinated Ti^4+^ centers) and, therefore, located on edges, steps and corners (Mino et al., 2012). All bands that are assigned to Ti^4+^—CO complexes undergo the expected frequency shift with the CO coverage, due to the building up of lateral–lateral interactions between CO oscillators adsorbed at the surface of TiO_2_ (Spoto et al., 1990; Signorile et al., 2015).

Along with the CO interaction with the MoS_2_/TiO_2_ surface (Figure 8B), it is noteworthy that, besides a general decrease in intensity of the main features with respect to TiO_2_, the main relevant effects of the sulfidation reaction of MoO_x_ at 673 K are the formation of the bands in the 2,130–2,000 cm^−1^ range. It is noteworthy that such observed frequencies can be justified only by the interaction of CO with Mo^x+^ species in reduced states (*x* < 4), which are presumably located at the edges of the MoS_2_, and that such system looses sulfur under high vacuum conditions at 673 K (Cesano et al., 2011). In particular, the main phenomena are the formation of a bandwith maximum at 2,109 cm^−1^, well evidenced at low CO coverages, and the developing of the broad band at 2,066 cm^−1^, which shifts to 2,070 cm^−1^ at decreasing CO pressure. More in detail, the 2,109 cm^−1^ and the 2,066 cm^−1^ bands have been ascribed to CO adsorbed on the defective sites located at the edges and corners of MoS_2_ slabs, as confirmed by some authors (Tsyganenko et al., 2004; Wu et al., 2004; Cesano et al., 2011). These two bands are by far the last ones that disappear by outgassing. In particular, the band at 2,109 cm^−1^ has been previously assigned to Mo^x+^ located on the edges, including Mo oxysulfide species (MoO_x_S_y_ phase) (Signorile et al., 2015), while the one at 2,066 cm^−1^ is due to reduced Mo^x+^ species associated with surface sulfur vacancies located on very exposed sites (Cesano et al., 2011; Cravanzola et al., 2017). Despite the relevant lattice mismatching between MoS_2_ and TiO_2_ (Dai et al., 2016), the Mo oxysulfide species observed in the aforementioned paper, by adopting the same experimental setup and conditions, are responsible for the good grafting of MoS_2_ at the surface of TiO_2_ nanoparticles. Owing to interfacial bonding between MoS_2_ and TiO_2_ (110), formation of MoS_2_ either in parallel or perpendicularly oriented shape to the rutile TiO_2_ (001) direction has been observed (Kibsgaard et al., 2009). Furthermore, Density Functional Theory (DFT) calculations show a favorable epitaxial relation, between the MoS_2_ edge sites and (101) anatase facelets, thus helping in the bonding of MoS_2_ even on anatase TiO_2_ (Arrouvel et al., 2005). It is worthy noticing that, by comparing the spectral evolution of CO adsorbed on the MoS_2_/TiO_2_ surface with that of CO adsorbed on the MoO_x_/TiO_2_ surface (Supplementary Figure 1), the intensity of 2,179 cm^−1^ peak is somehow restored. Even so, the 2,189–2,182 cm^−1^ envelope of MoO_x_/TiO_2_ is very low in intensity and is, as far as it goes, related to the wetness impregnation, causing the surface of TiO_2_ being decorated with highly dispersed Mo particles, thus interrupting the regularity of the exposed TiO_2_ (101) faces, which are usually responsible for increased absorptions. It can be hypothesized that, acting as a reactant, H_2_S is responsible for the formation of MoS_2_ domains from Mo cations and that the TiO_2_ (101) faces become again available to bond CO molecules.

Moving to the hydroxyl group region, the wide band observed in the 3,400–3,200 cm^−1^ interval, before CO dosage (inset of Figure 8B), is associated with the oxygen-sulfur exchange and with the formation of water at the surface. As reported in literature (Chen et al., 1999; Travert et al., 2002), O^2−^ can react with H_2_S to form SH^−^ and S^2−^ ions through heterolytic dissociation and water is formed at the surface, whose concentration is increasing with the amount of adsorbed H_2_S. Anyway, H_2_S molecules could interact also with surface –OH groups (bonded to Ti^4+^ or Mo^x+^), by means of hydrogen bonds (Jain et al., 2014). After CO adsorption, the wide band observed in the 3,400–3,200 cm^−1^ region is decreasing in intensity, probably due to the removal of the H_2_O molecules by the CO, together with the shift to a lower frequency of the broad and intense feature at 3,562 cm^−1^, as before discussed.

Along with the FTIR spectra of CO adsorbed at 77 K on *p*-C_2_H_2_/MoS_2_/TiO_2_, after the oligomerization step, some important considerations can be noticed (Figure 8C).

Firstly, when compared to the spectra of CO adsorbed on MoS_2_/TiO_2_, the main point is the complete erosion of the band at 2,179 cm^−1^, related to the interaction of CO molecules with TiO_2_ (101) surfaces. Lastly, the band at 2,140 cm^−1^ has grown noticeably in intensity, while the bands in the 2,170–2,150 cm^−1^ range, associated to CO adsorbed on other anatase faces, are still visible, even if with different intensities. This behavior can be described with the prevailing formation of the polymer on (101) faces of TiO_2_, even if its presence on the other faces is not negligible. This means that, after the oligomerization reaction, Ti sites located on different facelets are still available, but to a less extent than on MoS_2_/TiO_2_. Conversely the peak at 2,146 cm^−1^, owing to CO adsorption on rutile phase, has disappeared.

It is noteworthy that the weak peaks at 2,066, 2,070, and 2,109 cm^−1^, related to the adsorption of CO on MoS_2_, are absent in *p*-C_2_H_2_/MoS_2_/TiO_2_. This means that the species coming from C_2_H_2_ oligomerization, as far as possible, mask the Mo sites, making them no more available for interaction with the CO molecule.

Some more, as far as the bands of OH groups are concerned (inset of Figure 8C), no remarkable frequency shift and/or intensity change can be observed. We can hypothesize that OH groups are covered by the polymer and then they are no more available for CO interaction.

On the basis of these results, we can highlight that not only the Ti sites, but also the Mo ones could be involved in the oligomerization process of C_2_H_2_.

## Conclusions

The oligomerization of C_2_H_2_ was performed on a MoS_2_/TiO_2_ support. The MoS_2_/TiO_2_ system has been synthesized by dosing H_2_S at 673 K on TiO_2_, previously impregnated with ammonium heptamolybdate, used as a precursor of molybdenum species. By investigating the obtained samples by XRD and HRTEM, their crystalline structure and morphology have been discussed. In particular, the XRD patterns of MoS_2_/TiO_2_ reveal the formation of thin MoS_2_ at the surface of TiO_2_. This fact is confirmed by HRTEM images, where MoS_2_ particles have been found to have thicknesses ranging from 1 to 3 layers. Precious information on the oligomerization effects of C_2_H_2_ comes from Raman spectroscopy, by comparing MoS_2_/TiO_2_ system before and after C_2_H_2_ dosage. The spectrum of *p*-C_2_H_2_/MoS_2_/TiO_2_ has been interpreted, sure enough, as characterized by the typical Raman active modes of both, polyenic and aromatic domains.

Along with the reaction products, conjugated double bonds have been confirmed also by UV-vis spectra, while from FTIR results both polyenes and polycyclic aromatic hydrocarbons can be identified. Surprisingly, the aromatic species are favored when the C_2_H_2_ oligomerization reaction occurs at 298 K, while at higher temperatures the polyenes seem to be predominant. In principle, by following the bottom up approach, the *in situ* formation and growth of polyenes and/or graphene-like structures could be suitably tailored by controlling the experimental conditions (i.e., temperature, contact time, pressure). Moreover, from the analysis of FTIR spectra of CO adsorbed at the surface of samples, it has been safety concluded that both Ti and Mo sites play a key role as catalysts in the formation of unsaturated and poly-condensed aromatic carbon systems. In point of fact, both titanium and molybdenum (even if at low concentration) sites, are simultaneously involved in the C_2_H_2_ oligomerization reactions, thus giving rise to an uniform dispersion of the obtained structures, which are strongly anchored at the MoS_2_/TiO_2_ surface.

According to our results, by tailoring the structure and the interface of well-defined hybrid materials, new photoactive and catalytic systems with enhanced activity can be designed. The formation of large condensed rings, in effect, could represent a step toward the *in-situ* creation of a graphene layer and/or polyconjugated conductive species at the surface of a photoactive material.

Further work is needed to give insights about the nature of the products and the whole sequence of reactions entailed in the interaction of acetylene with MoS_2_/TiO_2_ systems. Nevertheless, the present study is aiming to follow a molecular self-assembly approach to nanomaterials, thus representing a step toward the direct production of graphene-like species or polyacetylene chains, both interesting for new and advanced applications.

## Author contributions

SC, FC, and FG conceived, designed and performed the experiments and characterizations, analyzed the data; SC, FC, and DS wrote the manuscript. All authors read and approved the paper.

### Conflict of interest statement

The authors declare that the research was conducted in the absence of any commercial or financial relationships that could be construed as a potential conflict of interest.
