# Supplementary material for: Carbon Domains on MoS2/TiO2 System via Catalytic Acetylene Oligomerization: Synthesis, Structure, and Surface Properties
Source: Front Chem. 2017 Nov 8;5:91. doi: 10.3389/fchem.2017.00091 (PMC5701632; doi:10.3389/fchem.2017.00091)
Supplement: Supplementary file 1 [file Presentation1.PDF]

Supplementary Material

Carbon domains on MoS_2/TiO_2 system via catalytic acetylene oligomerization: synthesis, structure and surface properties

Sara Cravanzola, Federico Cesano*, Fulvio Gaziano, Domenica Scarano

*** Correspondence:** Corresponding Author: federico.cesano@unito.it

a) Infrared spectra of CO adsorbed at the surface of MoO_x/TiO_2, at decreasing coverage (77 K). It is worth noticing that the bands in the 2189–2182 cm^{-1} range are very low in intensity (Supplementary Figure 1).

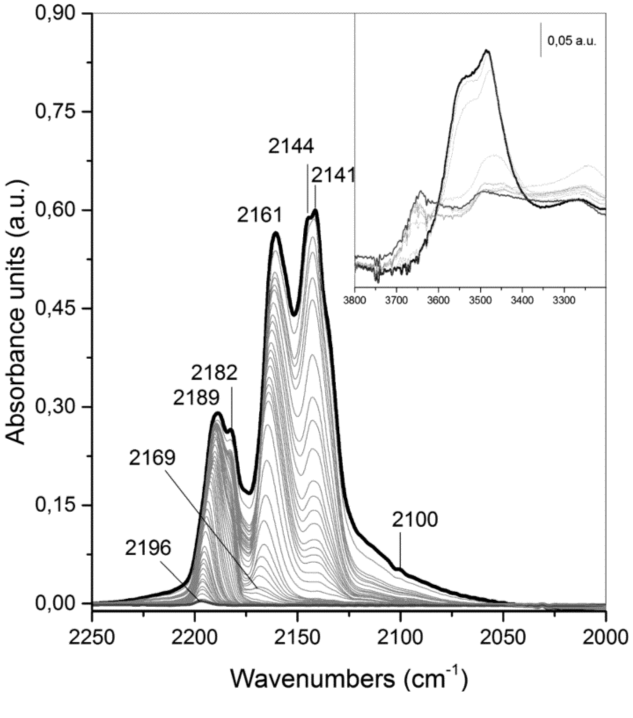

Supplementary Figure 1. IR spectra at 77 K of CO adsorbed on MoO_x/TiO_2. In the insert, the hydroxyl groups range is highlighted.

b) SEM acquisitions were performed on TiO_2 (P25), MoS_2/TiO_2 and *p*-$C_2H_2/MoS_2/TiO_2$ samples (Supplementary Figure 2).

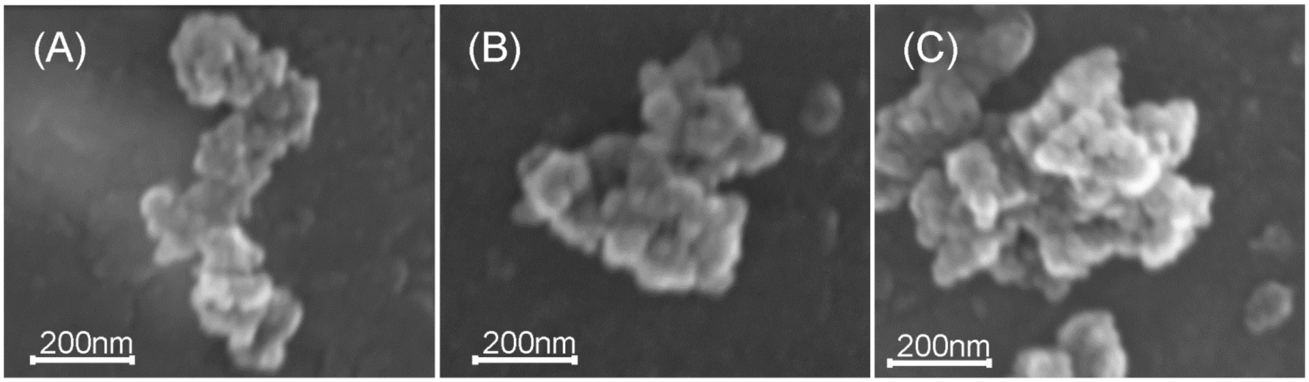

Supplementary Figure 2. SEM images of (A) TiO_2 (P25), (B) MoS_2/TiO_2 and (C) *p*-$C_2H_2/MoS_2/TiO_2$.

c) The evolution of the sample under the laser beam, during Raman acquisition (Supplementary Figure 3).

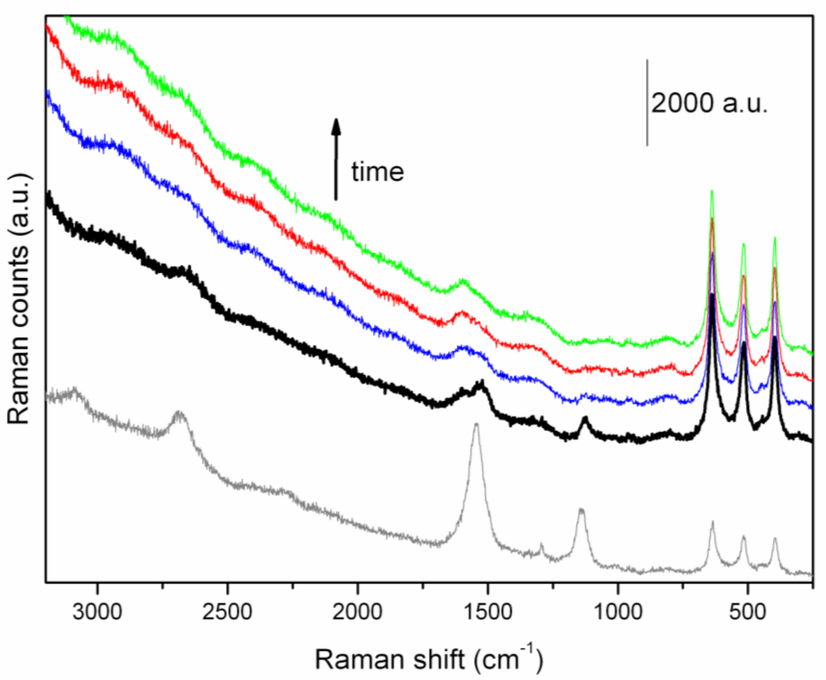

Supplemetary Figure 3. Raman spectra of *p*-$C_2H_2/MoS_2/TiO_2$ acquired with the 442 nm laser line. It can be observed the instability of the sample under the laser beam, for increasing exposition times (from black to green curve). The grey spectrum was acquired using a rotating configuration, to avoid the evolution phenomenon.
